# Chronic administration of atorvastatin could partially ameliorate erectile function in streptozotocin-induced diabetic rats

**DOI:** 10.1371/journal.pone.0172751

**Published:** 2017-02-28

**Authors:** Juhyun Park, Oh Seong Kwon, Sung Yong Cho, Jae-Seung Paick, Soo Woong Kim

**Affiliations:** 1 Department of Urology, Seoul Metropolitan Government Seoul National University Boramae Medical Center, Seoul, Korea; 2 Department of Urology, Hallym University Hangang Sacred Heart Hospital, Seoul, Korea; 3 Department of Urology, Seoul National University Hospital, Seoul, Korea; Chang Jung Christian University, TAIWAN

## Abstract

The efficacy of statins is related to the ‘common soil’ hypothesis, which proposes oxidative stress and inflammation as main pathophysiologic processes in the disease group of diabetes and endothelial dysfunction. This study evaluated the recovery of erectile function after administration of chronic statin alone in streptozotocin (STZ)-induced diabetes mellitus (DM) rats, focusing on the anti-oxidative effects and consequentially recuperated endothelial function. A total of 45 male Sprague-Dawley rats (8 weeks old) were divided into three groups (n = 15 each): an age-matched normal control group (Control group), an uncontrolled DM group (DM group), and a statin-treated group (Statin group). The rats in the DM and Statin group received an injection of STZ (60 mg/kg). Beginning 10 weeks after the establishment of DM, the Statin group received daily treatment with atorvastatin (10 mg/kg) via oral gavage for four weeks. After 14 weeks, the results of the experiment were evaluated. The ratios of intracavernosal pressure (ICP) to mean arterial pressure (MAP) were recorded with cavernosometry (20 Hz, 3 V, 0.2 msec for 30 seconds) before and after the intravenous administration of udenafil (1 mg/kg). Expression of alpha-smooth muscle actin (α-SMA) was evaluated using cavernosal tissue. In addition, changes in RhoA translocation ratio and myosin phosphatase target subunit 1 (MYPT1) phosphorylation were evaluated with western blot. Superoxide dismutase (SOD) and malondialdehyde (MDA) levels were also analyzed as measurements of oxidative stress levels. The ICP/MAP and area under the curve (AUC)/MAP ratios of the Statin group were obviously superior to the DM group, but were not comparable to the Control group (*P*<0.001). The level of oxidative stress, namely SOD activity, was also significantly lower in the Statin group than in the DM group (*P* = 0.015), and was comparable to the Control group. In contrast, MDA levels were not considerably different among the groups (*P* = 0.217). The RhoA translocation ratio was not significantly different among the groups (*P* = 0.668), whereas MYPT1 phosphorylation in the Statin group was significantly lower than in the DM group (*P* = 0.030), and similar to the Control group. Expression of α-SMA in the Statin group was higher than in the DM group (*P*<0.001), and comparable to the Control group. Chronic statin treatment alone showed anti-oxidative effects and helped to restore the erectile mechanism, but did not lead to the full recovery of erectile function in STZ-induced DM rats. Therefore, combination therapy rather than a single agent should be the preferred treatment strategy for DM-associated erectile dysfunction, especially in the setting of severe diabetes.

## Introduction

Diabetes mellitus (DM) is the most common cause of erectile dysfunction (ED) [1, 2]. In DM, increased reactive oxygen species (ROS) and decreased bioavailable nitric oxide induce endothelial dysfunction and atherosclerosis [3]. As DM progresses, the endothelium of the corpus cavernosum is also affected and undergoes irreversible structural damage, such as corporal fibrosis [4]. Finally, DM-associated erectile dysfunction (DMED) is an irreversible complicated pathophysiologic process, consequently, DMED patients suffer from a lower response rate to a phosphodiesterase type 5 inhibitor (PDE5i) than do patients with other functional types of ED [5].

The pathophysiologic mechanism of DMED includes both the functional and structural impairment. Endothelial dysfunction, coordination disorder of the relaxation and contraction of corporal smooth muscles via the nitric oxide (NO)/cyclic guanosine monophosphate (cGMP) pathway and the RhoA/Rho-kinase (ROCK) pathway, autonomic neuropathy and apoptosis in corporal smooth muscles were simultaneously happened. These processes do not work independently; diverse combinations of each pathologic mechanism may manifest as DM progresses [1, 6–9]. Therefore, to prevent and reinstate the endothelial function of the corpus cavernosum before progression of the irreversible changes is the best treatment option to overcome DMED.

In a previous study, we reported that conventional insulin treatment plus statins showed better efficacy than conventional insulin treatment alone at restoring endothelial function in DM rats, via inhibition of the ROCK pathway [7]. Even though, we did not include statin-only-treatment group in a previous study, we could expect statin-only treatment also considerably effective. Many researchers have reported that the efficacy of statin is related to the ‘common soil’ hypothesis, which proposes oxidative stress and inflammation as main pathophysiologic processes of the disease group of diabetes and endothelial dysfunction [9–11]. We supposed that the anti-oxidative effects of statin could be the most fundamental mechanism involved in the previous experimental results.

In the present study, we evaluated the recovery of erectile function after the administration of chronic statin alone in streptozotocin (STZ)-induced DM rats, in addition to the impact on the ROCK pathway, further analyzing the anti-oxidative effects and consequentially recuperated endothelial function.

## Materials and methods

### Experimental animals and design

A total of 45 male Sprague-Dawley rats (8 weeks old) were divided into three equal groups (n = 15 each): an age-matched normal control group (Control group), an uncontrolled DM group (DM group), and a statin-treated group (Statin group). Diabetes was induced by the intraperitoneal injection of 60 mg/kg STZ with a citrate phosphate buffer (50 mM sodium citrate, pH 4.5) after overnight fasting. The same volume of citrate buffer was administered to the Control group. All rats weighed 270–300 g before diabetes induction. DM was confirmed by blood glucose levels of >300 mg/dL 48 hours after the STZ injection, using an Accu-Chek Compact Meter (Roche Diagnostics, Indianapolis, IN, USA). All but five rats developed diabetes after a single injection of STZ. The remaining non-hyperglycemic rats received a second injection of 60 mg/kg STZ, and became diabetic within three days.

As the current experiment was aimed at restoring the erectile dysfunction and the analyzing the fundamental mechanism, the statin treatment was delayed until 10 weeks after the STZ injections. Our previous study revealed that the erectile function began to significantly decline 10–12 weeks after the onset of DM, which corresponded to the functional and structural impairment of the corpus cavernosum [8]. The Control and DM groups received no treatment, while the Statin group received daily administration of atorvastatin (10 mg/kg) via oral gavage. Atovastatin was purchased from Pfizer Ireland Pharmaceuticals (Dublin, Ireland). The purity of atovastatin was more than 99%. The dose of atorvastatin was determined to be similar to the effect of the statin in our previous study [7]. The statin treatment was continued for four weeks.

There were 15 rats in each group. In total, 45 rats were used in the experiment. None of the animals in the Control group died during the course of the experiment. However, four rats in the DM group died, and five rats in the Statin group died. The total mortality rate was 20%. When the rats were dying and showing the clinical sign such as dyspnea, cyanosis, low body temperature, and paralysis of one or more extremities, the rats were euthanized by the CO2 chamber.

On the day of the experiment, the rats were fasted overnight and then anesthetized with an intraperitoneal injection of Zoletil (50 mg/kg). Following measurement of body weight, all rats underwent *in vivo* cavernosometry. After cavernosometry, all rats were sacrificed by open pneumothorax. Then, a sample of blood was then obtained for serum HbA1c and blood glucose determinations. The cavernosal tissue was rapidly removed as a whole. The middle parts of the corpus cavernosum were cut out and kept overnight in 10% formaldehyde solution, then embedded for histological evaluation. Remnant tissue was promptly placed in liquefied nitrogen and stored at −80°C until further processing. The rats were continuously examined and monitored during the experiment, and there were no unexpected deaths in these cases. We used anesthesia to reduce the suffering and distress of animals before any process that is potentially stressful or painful.

All procedures were approved by the Institutional Animal Care and Use Committee of Seoul National University Hospital. All experimental and animal care rules were obeyed according to the National Research Council publication, the Guide for Care and Use of Laboratory Animals.

### *In vivo* assessment of erectile function

Cavernosometry was performed using the technique described in previous studies. [7, 8] Mean arterial pressure (MAP) was continuously monitored via the carotid artery, and intracavernosal pressure (ICP) was continuously observed via the corpus cavernosum after denuding the penile shaft. The major pelvic ganglion and cavernous nerve were exposed on the lateral side of the prostate, and a bipolar electrode was connected to an electrical stimulator (S48, Grass Instruments, Quincy, MA, USA). Electrical stimulation was performed with 2 V for 30 s at 0.2-ms intervals, at a frequency of 20 Hz. After checking the basal response, an intravenous bolus of udenafil, a novel PDE-5 inhibitor (1 mg/kg), was administered, and cavernosometry was performed again 1 min later. Pressure data were collected and analyzed electronically at every session (PowerLab, ADInstruments, Colorado Springs, CO, USA). The ratios of ICP to MAP and of area under the curve (AUC) to MAP were analyzed.

### *In vitro* assessment of structural changes

Immunohistochemical staining for alpha-smooth muscle actin (α-SMA) was carried out to evaluate the percentages of smooth muscle cell components (% α-SMA). Specimens were incubated overnight with primary anti-α-SMA antibody (1:100; Dako, Glostrup, Denmark) and processed with a biotin-labeled secondary antibody (1:2,000, Santa Cruz Biotechnology, Santa Cruz, CA, USA) for counterstaining. The ratio of α-SMA staining to total area was calculated under magnification (×25) in four duplicate sections of each sample with Image Pro Plus 4.5 software (Medica Cybernetics, Silver Spring, MD, USA).

### Western blot analysis

As described above, cavernosal tissue was obtained immediately after cavernosometry. The same quantities of protein extracts (20–50 μg) were separated in sodium dodecyl sulfate polyacrylamide gels, which were transferred to nitrocellulose membranes. Overnight incubation was carried out with primary antibodies. Anti-myosin phosphatase target subunit 1 (MYPT1) (1:2,000, Cell Signaling Technology), anti-phospho-MYPT1 (Thr696, 1:1,000, Millipore, Charlottesville, VA, USA), and mouse monoclonal anti-RhoA antibody (1: 1000; Cytoskeleton, Denver, CO, USA) were used as primary antibodies. Bands were visualized using an electrochemiluminescence (ECL) western blot development system (Pierce). To adjust for loading differences, the membranes were re-probed with an antibody against monoclonal anti-actin antibodies. Results were quantified using densitometry.

### Measurement of oxidative stress levels

Lipid peroxidation (malondialdehyde [MDA]) levels were measured in cavernosal tissue using an MDA assay kit (Abcam, Cambridge, MA, USA). Ten milligrams of tissue were homogenized on ice in 300 μl of MDA lysis buffer (Abcam), then centrifuged (13,000 × *g*, 10 min) to remove insoluble materials. Ten microliters of plasma were mixed with 500 μl of 42 mM H_2_SO_4_ and 125 μl of phosphotungstic acid solution at room temperature for 5 min. After centrifuging (13,000 × *g*, 3 min), the pellet was re-suspended on ice with 100 μl of double-distilled H_2_O. Then, 200 μl of solution and 600 μl of 2-thiobarbituric acid solution were incubated at 95°C for 60 min, before cooling to room temperature in the ice bath for 10 min. The intensity of absorbance at 532 nm was proportional to the MDA level.

Superoxide dismutase (SOD) activity was assessed in cavernosal tissues by a colorimetric assay kit (Abcam). The cell proliferation reagent utilized in the kit is a water-soluble tetrazolium salt (WST-1), which produces a water-soluble formazan dye that can be detected at 450 nm upon the reduction of WST-1 by superoxide anions. WST-1 reduction is inhibited by SOD, which catalyzes the dismutation of the superoxide anion to produce H_2_O_2_ and O_2_. Therefore, SOD activity was calculated on the basis of the percent inhibition of WST-1 reduction, which in turn reflected the percent inhibition of the superoxide anions.

### Statistical analysis

Data are shown as mean ± standard deviation. Differences in blood glucose levels and erectile parameters from the start of statin treatments were evaluated using analysis of variance (ANOVA) with post hoc Duncan’s and Tukey’s analyses or the Kruskal-Wallis test among more than three groups. A two-tailed *P* value of <0.05 was considered statistically significant for all statistical analyses. All statistical analyses were performed with commercially available statistical software (SPSS^®^ version 22.0; IBM, Armonk, NY, USA).

## Results

### Somatic growth and glycemic levels

Body weight, blood glucose levels, and HbA1c levels at the end of the experiment are summarized in Table 1. The rats in the Control group experienced constant weight gain, whereas the rats in the DM and Statin groups showed weight loss during the experimental period. Body weights were significantly different among the groups. Blood glucose levels in the DM and Statin groups were near the highest measurable level, while the Control group was within normal range. HbA1c levels showed a similar pattern. The DM and Statin groups showed significantly higher HbA1c levels than the Control group. There were no statistical differences between the DM and Statin groups in blood glucose and HbA1c levels.

**Table 1:** Body weight, serum glucose, and HbA1c levels at the end of the experiment.

Group	Control	DM	Statin
Weight (g)	486.6 ± 27.8 *^#^	163.8 ± 17.2 ^#^	203.2 ± 28.5 *
Blood glucose (mg/dL)	86.1 ± 4.8 *^#^	894.1 ± 17.7	870.6 ± 55.1
HbA1c (%)	4.0 ± 0.2 *^#^	8.2 ± 1.8	8.1 ± 1.9

Data presented as mean ± standard deviation

*Denotes statistical significance in comparison with the DM group (*P*<0.05)

^#^Denotes statistical significance in comparison with the Statin group (*P*<0.05)

### *In vivo* assessment of erectile function

**Fig 1:**
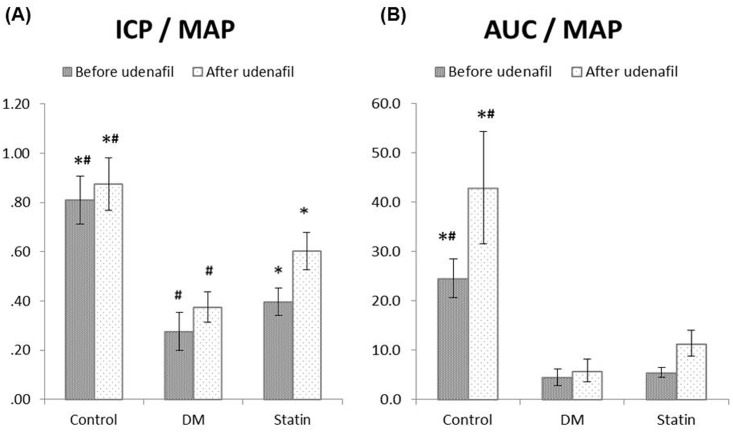
Comparison of erectile parameters of each group at 20 Hz, before and after intravenous administration of udenafil, a specific type 5 phosphodiesterase inhibitor. (A) Changes in ICP adjusted for MAP (ICP/MAP) and (B) changes in AUC adjusted for MAP (AUC/MAP). ICP, intracavernosal pressure; AUC, area under the curve; MAP, mean arterial pressure. *Denotes statistical significance in comparison with the DM group (*P*<0.05). ^#^Denotes statistical significance in comparison with the Statin group (*P*<0.05).

The peak ICP/MAP and AUC/MAP ratios with electrical stimulus before and after the administration of udenafil are described in Fig 1. Regardless of udenafil injection, the DM group had the lowest ICP/MAP ratio (*P*<0.001). The Statin group showed a significantly higher ICP/MAP ratio than the DM group, but it was not restored to the level of the Control group. On the other hand, AUC/MAP was lower in the DM and Statin groups than in the Control group, irrespective of udenafil administration. The Control group showed a statistical difference with the other two groups (*P*<0.001).

### *In vitro* assessment of structural changes

**Fig 2:**
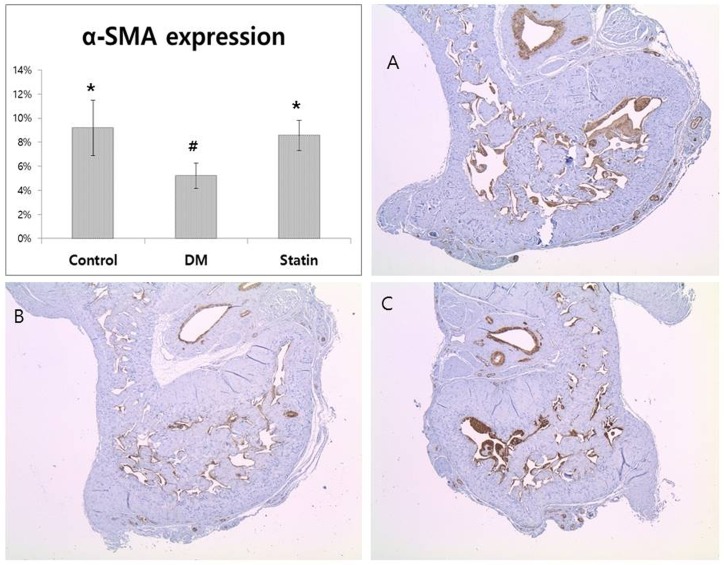
α-SMA expression in penile sections. Densitometric analysis and representative images for α-SMA immunohistochemical staining is shown. The percentages of smooth muscle cell component (% α-SMA) significantly decreased in the DM group (B) compared with the Control (A) and Statin (C) groups. α-SMA, alpha-smooth muscle actin. *Denotes statistical significance in comparison with the DM group (*P*<0.05). ^#^Denotes statistical significance in comparison with the Statin group (*P*<0.05).

Expression of α-SMA was significantly lower in the rats of the DM group than in the other groups (*P*<0.001). There was no statistical difference between the control and Statin groups (Fig 2).

### Western blot analysis

**Fig 3:**
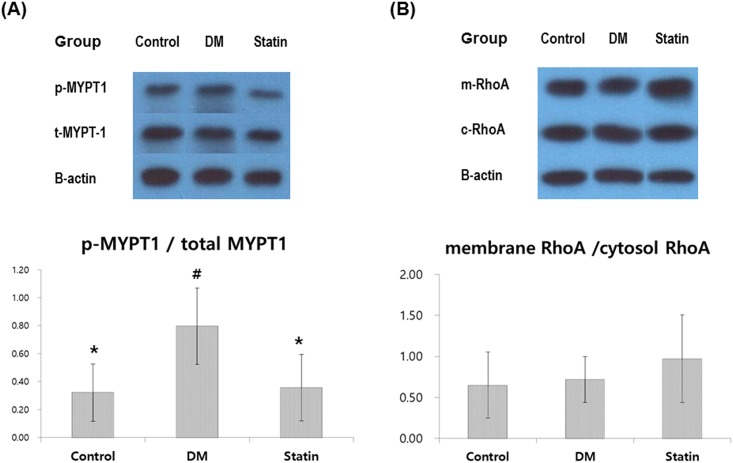
Western blot analyses for (A) phosphor-MYPT-1 and total MYPT-1 (B) RhoA (membrane) and RhoA (cytosol). ß-actin serves as a control. Representative blots are shown in the upper panel, and densitometric analyses are shown on the bottom. MYPT-1, phosphorylated myosin phosphatase target subunit 1; RhoA, Ras homolog gene family member A. *Denotes statistical significance in comparison with the DM group (*P*<0.05). ^#^Denotes statistical significance in comparison with the statin group (*P*<0.05).

A representative blot and densitometric analysis is described in Fig 3. Phosphorylation of MYPT1 in the DM group was significantly higher than in the other groups (*P* = 0.030). MYPT1 phosphorylation in the Control group was comparable to the Statin group. In contrast, there was no statistical difference among the groups with regard to the membrane/cytosol ratio of RhoA (*P* = 0.668, Fig 3).

### Measurement of oxidative stress levels

**Fig 4:**
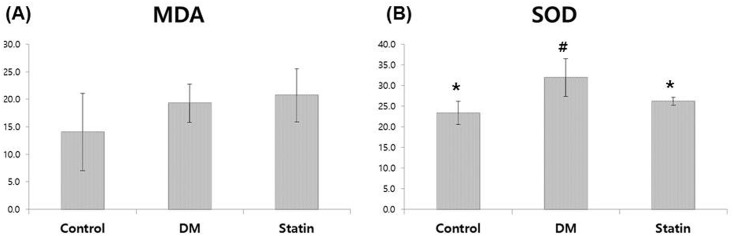
(A) Plasma MDA levels using the MDA assay kit. (B) Superoxide dismutase (SOD) activity with a colorimetric assay kit. MDA, malondialdehyde; SOD, superoxide dismutase. *Denotes statistical significance in comparison with the DM group (*P*<0.05). ^#^Denotes statistical significance in comparison with the Statin group (*P*<0.05).

In plasma MDA levels, there were no considerable differences among the groups (*P* = 0.217). On the other hand, SOD activity in the DM group was significantly higher than in the other groups (*P* = 0.015). The SOD activity of the Control group was comparable to that of the Statin group (Fig 4).

## Discussion

Statins show the characteristic pharmacological action of the metabolism of cholesterol, and also have various positive effects on endothelial function [12, 13]. Recently, several researchers have reported that chronic statin therapy in diabetic patients significantly decreased the incidence of cardiovascular disease and showed potential positive effects for the maintenance of endothelial function, regardless of the involvement of lipid metabolism [14–16]. In this respect, it is clear why some human studies have concluded that statin treatment has a beneficial effect on DMED [17–19].

These various forms of efficacy of statin therapy are associated with the ‘common soil’ hypothesis, which asserts that oxidative stress and inflammation are major pathophysiologic mechanisms in both diabetes and endothelial dysfunction [9–11]. We supposed that statins could inhibit oxidative stress and inflammation of the endothelium, and could furthermore prevent ED, which is one of the microvascular complications of DM.

As mentioned above, we reported that conventional insulin treatment plus statins showed better efficacy than conventional insulin treatment at restoring endothelial function in DM rats, via inhibition of the ROCK pathway; however, we did not include a statin-only treatment group in that study, as it was thought to be less useful clinically [7]. However, the investigation of statin-only treatment, but not of insulin plus statin treatment, is expected to provide previously unknown and important basic knowledge about statins, and can lead to more effective combinations with other medications.

In the present study, we identified functional and structural partial restoration from DMED using chronic statin therapy alone. The recovery of erectile function in the Statin group was obviously superior to that of the DM group, but was not comparable to the Control group. Nevertheless, the level of oxidative stress, namely SOD activity, in the Statin group was also significantly lower than in the DM group, and was comparable to the Control group. MYPT1 phosphorylation in the Statin group was meaningfully lower than in the DM group, and was similar to the Control group. In contrast, MDA levels and RhoA translocation ratio were not significantly different among the groups. In conclusion, statin therapy alone could help some functional pathway to recover, but it was not effective at all pathways.

We considered these confusing data, especially for MDA levels and RhoA translocation ratio to be consequences of severe induced DM and the different vulnerabilities and pregnabilities of each pathway. To date, no study analyzed the vulnerability of the damaging pathway depending on the severity of DM. However, when Cho et al. reported the changes of erectile function according to the different DM stages of STZ-induced rats, protein expression of ROCK was distinguishable earlier than that of MYPT1 [8]. Cumaoğlu and his colleagues also published that anti-oxidative effects of statin treatment in STZ-induced rats differed depending on the type of tissue [20]. Those results suggested different vulnerabilities and pregnabilities for each pathway.

In this study, the level of diabetes in the rats was very severe. We had confirmed DM when blood glucose levels were >300 mg/dL 48 hours after STZ injection [7, 8], however, in this study, most of the rats actually exceeded 600 mg/dL at the first check after injection, and reached the testable upper limit of 900 mg/mL during the 14-week rearing period, and the resulting graph formed a plateau rather than a gentle curve.

In our previous study, we categorized significant differences in HbA1c according to the intensity of insulin treatment of the STZ-induced DM rats in experiments conducted over a 14-week period. In a study by Cho et al. in 2014 of an STZ-induced DM rat model, the blood glucose and HbA1c levels were 649 mg/dL and 9.2±1.0%, respectively in the 14th week of the experiment [21]. However, in this study, we could not identify a significant difference in the HbA1c levels between DM group and Statin group. The discord between the findings of the two studies might be due to the severity of DM, with the degree of DM more severe in the present study than in the previous one and the statin treatment increasing the bodyweight but decreasing insulin sensitivity. As a result, there was no difference in ambient blood glucose or hemoglobin A1C between the two STZ-induced DM rat groups [22]. In addition, the lack of concordance between the HbA1c level and expected value may be explained by the severe DM status, which could have affected red blood cell physiology [23].

Consequently, we were in a difficult situation, as the mortality rate of the study was high at approximately 20%, and the surviving rats failed to gain weight during the rearing period. In general, there have been no upper limits, only lower limits, for the confirmation of DM in the STZ-induced diabetic rat model [24]. Even with the same medication, the efficacy would vary according to the degree of DM; therefore, determining the tolerable range of DM severity could be an interesting theme for future studies.

Expression of α-SMA in penile sections is one of the convincing results representing the anti-oxidative effects of statin treatment. Increased vascular tone and relaxation of corporal smooth muscle are the key processes of normal erection. However, in STZ-induced DM rats, increased cavernosal oxidative stress, augmented protein oxidative damage, and decreased eNOS expression result in erectile dysfunction. Functional dysfunction, impairment of NO physiology and the ROCK pathway are considered reversible in early erectile dysfunction. In contrast, irreversibly decreased α-SMA caused by augmented oxidative damage of proteins and inflammatory processes are considered as permanent structural dysfunction [25].

In the chronic diabetic condition, oxidation of smooth muscle could result in inflammation and apoptosis. [26] However, in our study, statin treatment prevented the inflammatory process and reduced apoptosis; as a result, α-SMA expression was higher in the Statin group than in the DM group, and comparable to the Control group. The bodyweight was slightly increased or maintained following the statin treatment. Therefore, the structure of penile tissue was less damaged, and functions, such as the carvernosal response, were sustained. Park et al. reported similar results, finding that rats in a statin-treated group were significantly heavier than those in a non-treatment group [7]. The association of statins with antioxidant and anti-inflammatory pathways is well known [27]. The mechanisms underlying the activity of the statin may have helped to maintain structural and functional carvernosal responses.

We extrapolated that these positive findings in current study would be more powerful evidence of the anti-oxidative effects and erectile function recovery with statin treatment in DMED. Because DMED is a complication produced from interactions between diverse pathways rather than a simple result with an obvious causality, these research data would be rather persuasive. Chronic administration of statin could just partially recover of erectile dysfunction in severely induced diabetic rats. If human clinical study would be planned, the combination therapy such as PDE5i, insulin and statin, etc. rather than a single agent like as a statin-only treatment in this study should be the preferred treatment strategy for DMED and the patients should be classified according to the severity of diabetes.

The major limitation of our study is that we did not thoroughly investigate all of the pathophysiologic mechanisms involved in diabetes-related ED. Because severe DM was induced by STZ injection, and weight gain was poor in almost all the DM rats in this study, the amount of harvested cavernosal tissue was very small. Therefore, any tissue assay would have been limited. Consequentially, potential markers associated with antioxidant mechanisms were preferentially analyzed. It was difficult to perform an assay for examination of intracellular signaling. We focused on anti-oxidative mechanisms and inferred that the functional and structural partial restorations from DMED were the result of anti-oxidation due to statin treatment. The blockading of the ROCK pathway by insulin plus the statin treatment was one of the major findings from our previous study, but this was not identified in the current study. The ROCK pathway might be more vulnerable according to the severity of DM [8]. Furthermore, our results suggested different vulnerabilities and pregnabilities for each pathway of DMED. Nevertheless, we can be certain that DMED is the final results of irreversible complicated pathophysiologic process. Statin therapy alone is able to prevent only a part of this complex system, and combination therapy is more ideal as an aggressive treatment for DMED.

### Conclusion

Chronic statin treatment alone showed anti-oxidative effects and helped to restore the erectile mechanism, but did not lead to full recovery of erectile function in STZ-induced DM rats. Therefore, combination therapy such as PDE5i, insulin and statin, etc. should be the preferred treatment strategy for DMED, especially in the setting of severe diabetes.

## Supporting information

S1 Table Body weight and blood glucose level of rats during the experiment. (XLSX)

S2 Table Dataset of the experiment. (DOCX)

S1 Fig Unedited MYPT1 western blot. (JPG)

S2 Fig Unedited RhoA western blot. (JPG)
