# Supplementary material for: Chronic administration of atorvastatin could partially ameliorate erectile function in streptozotocin-induced diabetic rats
Source: PLoS One. 2017 Feb 28;12(2):e0172751. doi: 10.1371/journal.pone.0172751 (PMC5330475; doi:10.1371/journal.pone.0172751)
Supplement: S2 Table — (DOCX) [file pone.0172751.s002.docx]

**S2 Table. Dataset of experiment.**

|  | **Conrol** | **DM** | **Statin** |
| --- | --- | --- | --- |
| ICP/MAP ratio |  |  |  |
| Before Udenafil | 0.81 ± 0.10 | 0.28 ± 0.08 | 0.37 ± 0.05 |
| After Udenafil | 0.87 ± 0.11 | 0.37 ± 0.06 | 0.60 ± 0.08 |
| AUC/MAP ratio |  |  |  |
| Before Udenafil | 24.61 ± 3.95 | 4.49 ± 1.65 | 11.41 ± 2.58 |
| After Undenafil | 42.93 ± 11.41 | 5.82 ± 2.27 | 19.17 ± 17.51 |
|  |  |  |  |
| α-SMA expression (%) | 0.09 ± 0.02 | 0.05 ± 0.01 | 0.09 ± 0.01 |
|  |  |  |  |
| Western Blot |  |  |  |
| MYPT1 ratio | 0.32 ± 0.20 | 0.80 ± 0.27 | 0.36 ± 0.24 |
| RhoA ratio | 0.65 ± 0.40 | 0.72 ± 0.28 | 0.97 ± 0.53 |
|  |  |  |  |
| Oxidative Stress Level |  |  |  |
| MDA | 1.41 ± 0.70 | 1.93 ± 0.34 | 2.07 ± 0.48 |
| SOD | 23.35 ± 2.78 | 31.93 ± 4.53 | 26.15 ± 0.93 |
